# Food groups consumed by infants and toddlers in urban areas of China

**DOI:** 10.3402/fnr.v60.30289

**Published:** 2016-02-09

**Authors:** Pan Yu, Liya Denney, Yingdong Zheng, Gerard Vinyes-Parés, Kathleen C. Reidy, Alison L. Eldridge, Peiyu Wang, Yumei Zhang

**Affiliations:** 1 School of Public Health, Peking University, Beijing, China; 2 Department of Public Health Nutrition, Nestlé Research Centre, Lausanne, Switzerland; 3 Nestlé R&D Centre, Beijing, China; 4 Nestlé Nutrition Global R&D, Florham Park, NJ, USA

**Keywords:** MING, dietary patterns, infants, young children, China

## Abstract

**Background:**

Food consumption patterns of young children in China are not well known.

**Objective:**

Characterised food groups consumed by infants and young children in urban China using data from the Maternal Infant Nutrition Growth (MING) study.

**Design:**

One 24-h dietary recall was completed for 1,350 infants and young children (436 infants aged 6–11 months and 914 young children aged 12–35 months), who were recruited from maternal and child care centres in eight cities via face-to-face interviews with the primary caregiver. All foods, beverages and supplements reported were assigned to one of 64 food groups categorised into the following: milk and milk products, grains, vegetables, fruits, protein foods and desserts/sweets. The percentage of infants and young children consuming foods from specific food groups was calculated, regardless of the amount consumed.

**Results:**

Less than half of infants consumed breast milk (47%), whereas 59% of infants consumed infant formula and 53–75% of young children consumed growing-up (fortified) milk. Rice was the number one grain food consumed after 6 months (up to 88%) and the consumption of infant cereal was low. About 50% of infants did not consume any fruits or vegetables, and 38% of young children did not consume any fruits on the day of the recall. Only 40% of all children consumed dark green leafy vegetables and even fewer consumed deep yellow vegetables. Eggs and pork were the most commonly consumed protein foods.

**Conclusions:**

The data provide important insight for developing detailed food consumption guidelines for this population group. Mothers of infants should be encouraged to continue breastfeeding after the first 6 months. Parents should be advised to offer a wide variety of vegetables and fruits daily, particularly dark green leafy and deep yellow vegetables and colourful fruits. The consumption of fortified infant cereal should be advocated to improve the iron intake of Chinese infants.

Adequate nutrition during early life is vital for optimal growth and development (1). A convincing body of research evidence demonstrates that early nutrition and food consumption patterns have long-lasting effects on the risks of later obesity and non-communicable diseases, including type 2 diabetes, hypertension, and cardiovascular disorders (2–5). It is known that between birth and the third year, infants and young children have completed the dietary transition, from an all-milk diet to a varied diet of family foods (6, 7). During this transition period, infants and young children are susceptible to nutritional intakes that are insufficient to meet their bodies’ needs (8). In addition, these young children are exposed to a variety of novel foods for the first time and food preferences are gradually established (9–11). Previous studies have reported that food preferences and eating patterns developed in early childhood appear to continue into adolescence and adulthood (12–14).

Dietary patterns represent a general profile of food and nutrient consumption, characterised on the basis of the usual eating habits. Therefore, it is important to evaluate food consumption patterns beyond the nutrient intake data to better understand the relationship between food intake and the risk of disease (15–17). However, research on the food consumption patterns of infants and young children is limited. Until now, a number of studies have been conducted in the United States (USA) and several countries in Europe and South America (6, 18–22), which provided important insight into the food consumption habits of young children in those countries. In China, most studies conducted on infants and young children have focussed on infant feeding practices, the timing of the introduction of complementary foods or feeding behaviours (23–26). Little has been reported on the food consumption patterns of young children.

The aim of this study was to characterise the food groups consumed by infants and young children who were recruited from maternal and child care centres (MCCC) in eight cities in China using the data from the Maternal Infant Nutrition Growth (MING) study.

## Subjects and methods

### Subjects

A total of 1,350 infants and young children, aged 6–35 months, participated in the MING study and were included in the present analysis. The study design and subject recruitment of the MING study have been described previously (27). Briefly, the MING study was a cross-sectional study to investigate the dietary and nutritional status of pregnant women, lactating mothers, and infants and young children whose age ranged from birth to 35 months. The infants and young children were recruited from MCCC in eight cities in China, based on child registration information. Infants and young children aged 6–35 months were randomly selected according to their age. The primary caregivers of the young children were approached for recruitment. The response rate was 66%. In this study, children under 12 months of age were called infants, and children 12–35 months of age were called young children.

The study was conducted according to the guidelines in the Declaration of Helsinki. All of the procedures involving human subjects were approved by the Medical Ethics Research Board of Peking University (No. IRB00001052-11042). Written informed consent was obtained from the primary caregiver of each infant or young child participating in the study.

### Data collection

One 24-h dietary recall was collected from the primary caregiver of each child via face-to-face interviews by trained interviewers. The interviewer asked the primary caregiver about all food, beverages, and supplements that the child consumed on the previous day. To make the results of this study comparable with the information obtained in other countries, a list of 64 food groups was categorised into the following: milk and milk products, grains, vegetables, fruits, protein foods, and desserts/sweets. The groupings were based on similarities between the nutrient content and dietary role identified in the categories and foods used in the studies of other countries. The classifications of food and food groups were similar to those used by Fox et al. (20, 28) but adapted to reflect the particular characteristics of the Chinese diet by including traditional, frequently consumed foods, such as noodles, steamed bread, and Chinese cabbage.

Fortified milk power produced specifically for young children aged 1–3 years is commonly available in China and is called ‘growing-up milk’ in this study (a direct translation from the Chinese name). More detailed information about dietary data collection, data handling, and food group classification has been previously described (27, 29).

### Analytic methods

The food group classifications were used to calculate the percentage of infants and young children who consumed specific foods or food groups at least once on the day of the 24-h dietary recall, regardless of the amount consumed. This method has been previously used by Fox et al. (20, 21, 30). In addition, the average amount consumed from the top five foods in each food category (per capita) is also reported. All estimates were calculated using the Statistical Analysis Software (Version 9.2, 2008, SAS Institute, Inc., Cary, NC). In order to understand food consumption patterns in detail, as well as differences among ages, the percentage of infants and young children who consumed specific foods or food groups at least once on the day of recall and the average amount consumed is presented for eight age groups: 6–8, 9–11, 12–14, 15–17, 18–20, 21–23, 24–29, and 30–35 months (20).

## Results

### Population characteristics

Among the infants and young children, 54.6% were boys and 45.4% were girls. Ninety percent of the mothers were 34 years of age or younger when their babies were born. About 79% of the mothers completed high school or a higher level of education. About half of the families had a monthly household income (per capita) that fell at or below the RMB (Reminbi, Chinese currency) 2,001–3,000 category and about half of the families had a monthly household income higher than the RMB 2,001–3,000 category. This income category covers the average income RMB 2,047 for urban households in all regions in China and the average income 2,726 RMB for urban households in the developed, eastern regions of China (31). Sixty percent of the children were cared for by mothers and 37% by grandparents.

### Milk

The percentage of children consuming any milk was above or slightly below 90% across all age groups (Table 1). Fifty-eight percent of infants were still breastfeeding at 6–8 months. The proportion decreased sharply to 34% at 9–11 months and decreased further after 12 months, with almost no children being breastfed by the end of 23 months. The average amount of milk consumed in each age group, in descending order by the amount consumed, is shown in Table 2.

**Table 1:** Percentage of infants and young children consuming different types of milk at least once a day in the Maternal Infant Nutrition Growth (MING) study

	Age group							
	
Food/food groups	6–8 months (*n =* 201)	9–11 months (*n =* 235)	12–14 months (*n =* 125)	15–17 months (*n =* 75)	18–20 months (*n =* 160)	21–23 months (*n =* 110)	24–29 months (*n =* 248)	30–35 months (*n =* 196)
Any milk	94	91	94	93	89	88	88	88
Breast milk	58	35	18	11	5	2	0	1
Infant formula	60	58	3	1	1	6	2	1
Growing-up milk	10	12	73	73	75	71	60	53
Cow's milk	2	4	12	17	15	19	34	46
Soy milk	0	0	0	0	1	1	0	0

**Table 2:** Average amount of the top five milk, grain, and protein sources consumed by children in the Maternal Infant Nutrition Growth (MING) study

	Milk		Grain and grain product		Protein sources	
	
	Food	Per capita ml/day (mean±SE)	Food	Per capita g/day (mean±SE)	Food	Per capita g/day (mean±SE)
6–8 months (*n =* 201)	Breast milk	263.0±18.6	Rice	28.7±4.8	Eggs	24.1±3.6
	Infant formula	40.5±3.3	Infant cereal	17.1±2.4	Pork/ham	5.0±1.3
	Cow's milk	3.6±2.5	Noodle	9.5±2.6	Dried beans/meat substitutes	2.6±1.2
	Growing-up milk	1.7±0.8	Steamed bread	2.7±0.8	Fish/shrimp/shellfish	2.4±1.6
	–		Millet	1.1±0.5	Organ meat	0.6±0.3
9–11 months (*n =* 235)	Breast milk	180.3±17.0	Rice	63.1±6.5	Eggs	32.6±2.6
	Infant formula	41.6±3.2	Noodle	25.4±4.0	Pork/ham	7.5±1.2
	Cow's milk	16.6±6.3	Infant cereal	10.1±1.9	Fish/shrimp/shellfish	5.2±1.1
	Growing-up milk	2.1±0.8	Millet	5.6±1.8	Dried beans/meat substitutes	3.2±0.9
	–		Steamed bread	5.2±1.2	Organ meat	0.8±0.3
12–14 months (*n =* 125)	Cow's milk	53.1±19.0	Rice	78.8±10.1	Eggs	33.9±3.1
	Growing-up milk	47.3±5.5	Noodle	30.2±6.3	Pork/ham	12.3±2.4
	Breast milk	36.3±7.9	Infant cereal	5.2±2.0	Dried beans/meat substitutes	8.1±3.3
	Infant formula	1.1±0.5	Millet	4.3±1.4	Fish/shrimp/shellfish	6.4±1.8
	–		Steamed bread	3.5±1.1	Chicken/duck	3.5±1.3
15–17 months (*n =* 75)	Cow's milk	87.7±24.8	Rice	102.3±12.7	Eggs	37.7±4.7
	Growing-up milk	57.4±9.0	Noodle	31.9±6.0	Pork/ham	11.1±2.8
	Breast milk	26.1±10.1	Millet	12.8±4.4	Fish/shrimp/shellfish	7.2±2.4
	Infant formula	0.3±0.3	Steamed bread	8.7±2.6	Dried beans/meat substitutes	5.8±1.9
	–		Infant cereal	1.8±1.5	Chicken/duck	1.4±0.8
18–20 months (*n =* 160)	Growing-up milk	42.8±4.0	Rice	106.7±9.0	Eggs	40.3±3.4
	Cow's milk	42.2±9.0	Noodle	24.6±3.8	Pork/ham	24.3±3.0
	Breast milk	8.3±3.3	Bread	11.1±0.9	Fish/shrimp/shellfish	20.0±6.2
	Infant formula	1.3±0.8	Steamed bread	6.2±1.5	Dried beans/meat substitutes	12.0±2.8
	–		Millet	3.8±1.1	Chicken/duck	6.3±2.2
21–23 months (*n =* 110)	Cow's milk	72.3±15.0	Rice	95.3±9.3	Eggs	41.6±4.0
	Growing-up milk	44.6±5.0	Noodle	27.9±5.2	Pork/ham	32.4±4.4
	Infant formula	3.6±1.3	Steamed bread	7.4±1.7	Dried beans/meat substitutes	12.1±2.8
	Breast milk	1.6±1.1	Cornmeal	5.5±3.8	Fish/shrimp/shellfish	6.0±1.9
	–		Millet	4.5±1.5	Chicken/duck	4.4±1.8
24–29 months (*n =* 248)	Cow's milk	94.6±9.8	Rice	109.3±6.8	Eggs	41.9±2.7
	Growing-up milk	32.5±3.2	Noodle	30.9±4.0	Pork/ham	26.4±2.8
	Infant formula	0.7±0.3	Bread	13.3±0.8	Dried beans/meat substitutes	23.0±6.2
	Breast milk	0.5±0.5	Steamed bread	7.4±1.5	Fish/shrimp/shellfish	13.1±2.2
	–		Millet	3.0±0.7	Chicken/duck	7.0±1.8
30–35 months (*n =* 196)	Cow's milk	122.1±12.5	Rice	111.1±10.0	Eggs	48.7±3.1
	Growing-up milk	23.2±2.2	Noodle	29.5±3.6	Pork/ham	33.2±3.0
	Breast milk	0.5±0.4	Bread	18.1±1.3	Dried beans/meat substitutes	30.0±5.7
	Infant formula	0.4±0.2	Millet	7.0±2.1	Fish/shrimp/shellfish	15.5±3.3
	–		Steamed bread	4.9±1.3	Beef	9.1±1.7

Infant formula was commonly consumed among infants with an average proportion of 59%. Forty-six percent of the infants who were breastfed were also given infant formula. Growing-up milk was the main source of milk among young children with a proportion of more than 50% across age groups. Thirty-five percent of young children aged 12–17 months who were breastfed were also given growing-up milk. The highest period of growing-up milk consumption was between 12 and 23 months with an average proportion of 75%. The proportion of children consuming cow's milk was very low among infants. It increased to 12–19% between 12 and 23 months and then reached to 46% among the oldest young children. Soy milk was rarely consumed in this study population.

### Grain and grain products

Nearly all infants and young children over the age of 9 months consumed some kind of grain product (Table 3). At age 6–8 months, about 40% consumed infant cereal but the proportion dropped sharply to 26% at age 9–11 months and then to 9% at age 12–14 months. From 6 months of age, rice remained the predominant grain-based food among all infants and young children, with 69% consuming rice at 9–11 months and 78–88% consuming rice between 12 and 35 months. Noodles, another predominant grain food consumed by children in all age groups, were consumed by 32% of infants 9–11 months. The proportion fluctuated from 33 to 43% among young children. Other grain products consumed included steamed bread, millet, bread and crackers. The average amount of the top five grain sources consumed in each age group is shown in Table 2.

**Table 3:** Percentage of infants and young children consuming different types of grain at least once a day in the Maternal Infant Nutrition Growth (MING) study

	Age group							
	
Food/food groups	6–8 months (*n =* 201)	9–11 months (*n =* 235)	12–14 months (*n =* 125)	15–17 months (*n =* 75)	18–20 months (*n =* 160)	21–23 months (*n =* 110)	24–29 months (*n =* 248)	30–35 months (*n =* 196)
Any grain or grain products	87	97	98	99	100	97	99	99
Rice^a^	44	69	78	80	86	81	88	85
Infant cereals	40	26	9	4	3	2	4	8
Noodles	16	32	37	43	33	41	34	42
Steamed bread	10	13	10	20	14	20	15	11
Millet	4	12	12	15	9	12	10	11
Bread	2	5	6	4	8	4	9	11
Pancakes^b^	1	0	2	0	3	4	4	6
Cornmeal	1	1	3	4	4	5	2	5

a Includes steamed rice, rice porridge, sticky rice, and non-infant rice noodles.

b Includes pancakes, fried bread stick, seedcake, and clay oven rolls.

### Fruits

At age 6–8 months, only about 48% consumed fruits (including 100% fruit juice) at least once a day (Table 4). After 9 months of age, the proportion of children consuming fruit fluctuated from 55 to 73%. Although fruit consumption improved slightly after 9 months of age, about 30–50% of children 9–35 months old did not consume any fruits on the day of the recall. In this population, 100% fruit juice was rarely consumed.

**Table 4:** Percentage of infants and young children consuming different types of fruits at least once a day in the Maternal Infant Nutrition Growth (MING) study

	Age group							
	
Food/food groups	6–8 months (*n =* 201)	9–11 months (*n =* 235)	12–14 months (*n =* 125)	15–17 months (*n =* 75)	18–20 months (*n =* 160)	21–23 months (*n =* 110)	24–29 months (*n =* 248)	30–35 months (*n =* 196)
Any fruit or juice	48	55	62	55	68	73	60	63
Any fruit	46	53	60	52	67	73	59	63
Types of non-baby food fruits								
Canned fruit	0	0	0	1	0	0	0	0
Any fresh fruit	46	53	59	52	66	72	59	62
Any dried fruit	0	0	3	3	5	4	3	3
Types of fruit^a^								
Apple	29	35	29	32	38	32	33	30
Banana	12	14	17	15	19	12	13	14
Citrus fruits	9	15	27	19	24	31	26	28
Pear	4	2	6	5	6	10	7	8
Grapes	3	3	2	4	4	5	2	0
Kiwi	1	3	4	3	8	4	2	3
Peach	2	2	2	5	3	2	1	2
Melon	2	0	2	1	3	2	2	1
Berries^b^	1	1	2	1	3	2	2	1
Others^c^	2	3	4	5	4	6	6	7
Baby food fruits	1	1	0	0	0	0	0	0
100% fruit juice								
Apple juice	2	1	2	3	1	0	1	1
Orange juice	0	0	0	0	0	0	0	0

a Includes non-baby food fruits.

b Includes strawberries, cherry, and mulberry.

c Includes persimmon, pomegranate, longan, dragon fruits, jujube, pineapple, plum, litchi, and mango.

Fresh fruits were the predominant type of fruit consumed in this population (Table 4). Apples were reported most often, followed by bananas and citrus fruits, which were also frequently reported across all age groups. Other fruits, including pear, peach, grapes and kiwi, were among the top five fruits reported. The average amount of the top five fruits consumed in each age group is shown in Table 5.

**Table 5:** Average amount of the top five fruits and vegetables consumed by children in the Maternal Infant Nutrition Growth (MING) study

	Fruits	Per capita g/day (mean±SE)	Vegetables	Per capita g/day (mean±SE)
6–8 months (*n*=201)	Apple	18.9±3.3	Chinese cabbage	3.1±0.9
	Banana	4.9±1.2	Carrots	2.7±0.9
	Citrus fruits	1.9±0.5	Spinach	2.6±1.5
	Pear	4.5±3.1	Cole	0.9±0.4
	Grapes	0.5±0.2	Sweet potato	0.9±0.4
9–11 months (*n*=235)	Apple	29.8±3.6	Chinese cabbage	4.8±1.1
	Citrus fruits	8.0±1.7	Spinach	4.2±1.3
	Banana	8.3±1.8	Carrots	2.7±0.7
	Grapes	0.6±0.2	Tomato	4.6±1.5
	Kiwi	2.6±1.1	Cole	1.7±0.4
12–14 months (*n*=125)	Apple	21.0±4.0	Chinese cabbage	7.8±1.6
	Citrus fruits	19.6±3.9	Spinach	8.1±4.1
	Banana	12.3±2.7	Tomato	4.6±1.3
	Pear	0.9±4.1	Carrots	4.5±2.0
	Kiwi	2.7±1.3	Cole	3.4±1.2
15–17 months (*n*=75)	Apple	29.9±6.4	Chinese cabbage	8.8±2.8
	Citrus fruits	13.6±4.5	Tomato	7.0±2.2
	Banana	10.6±3.5	Spinach	4.6±1.7
	Pear	9.7±4.7	White potato	3.5±1.8
	Peach	8.8±5.6	Carrots	4.6±2.0
18–20 months (*n*=160)	Apple	36.2±4.8	Chinese cabbage	13.9±2.3
	Citrus fruits	20.0±3.9	Spinach	4.9±1.2
	Banana	15.7±3.2	Tomato	6.8±2.4
	Kiwi	11.8±4.5	White potato	3.8±1.1
	Pear	4.3±1.8	Cole	3.4±1.1
21–23 months (*n*=110)	Apple	31.0±5.7	Chinese cabbage	15.5±3.2
	Citrus fruits	20.4±4.1	Tomato	5.5±1.9
	Banana	9.5±3.0	Spinach	4.5±1.4
	Pear	10.3±3.3	White potato	3.7±1.2
	Grapes	0.7±0.3	Cole	3.0±1.2
24–29 months (*n*=248)	Apple	36.2±7.5	Chinese cabbage	12.7±2.6
	Citrus fruits	23.1±3.8	Carrots	6.3±1.1
	Banana	13.9±2.9	Tomato	8.2±1.6
	Pear	7.5±2.4	Cole	8.5±1.9
	Grapes	0.8±0.4	Spinach	7.9±2.0
30–35 months (*n*=196)	Apple	27.3±3.9	Chinese cabbage	22.3±2.7
	Citrus fruits	29.1±5.1	Carrots	9.7±1.6
	Banana	15.0±3.7	Tomato	7.9±1.5
	Pear	7.8±2.3	Nori	5.4±1.2
	Kiwi	2.5±1.0	Cole	7.3±1.8

### Vegetables

About 37% of infants consumed some kind of vegetable at age 6–8 months and 57% at age 9–11 months (Table 6). After 12 months of age, the percentage of children consuming vegetables continued to rise and reached nearly 91% by age 30–35 months.

**Table 6:** Percentage of infants and young children consuming different types of vegetables at least once a day in the Maternal Infant Nutrition Growth (MING) study

	Age group							
	
Food/food groups	6–8 months (*n =* 201)	9–11 months (*n =* 235)	12–14 months (*n =* 125)	15–17 months (*n =* 75)	18–20 months (*n =* 160)	21–23 months (*n =* 110)	24–29 months (*n =* 248)	30–35 months (*n =* 196)
Any vegetable	37	57	77	71	83	84	87	91
Types of non-baby food vegetables^a^								
Dark green vegetables^b^	11	22	34	31	34	31	38	31
Deep yellow vegetables^c^	11	17	18	21	10	16	26	35
White potatoes	2	5	6	11	10	9	13	10
French fries and other fried potatoes	1	0	1	1	2	4	2	2
Other starchy vegetables^d^	3	4	6	7	8	9	18	24
Other vegetables^e^	17	32	55	52	64	63	65	76
Baby food vegetables	3	1	1	0	0	0	0	1

a Includes non-baby food vegetables.

b Dark green vegetables include broccoli, spinach, cole, Chinese chives, and romaine lettuce.

c Deep yellow vegetables include carrots, pumpkin, and sweet potatoes.

d Starchy vegetables include corn, green peas, broad beans, Chinese yam, lotus root, and taro.

e Other vegetables include Chinese cabbage, green beans, celery, peppers, mushrooms, eggplant, cucumber, tomatoes/tomato sauce, cauliflower, yellow beans (soy beans), zucchini, onions, lettuce, garlic bolt, agaric, garlic sprouts, white radish, nori, wax gourd, kelp, bitter gourd, loofah, green onion, lily, and pickles.

However, the percentage of children consuming dark green leafy vegetables was generally low, ranging from 11 to 22% among infants and 31 to 38% among young children. The proportion of children consuming deep yellow vegetables was even lower than the proportion consuming dark green vegetables, except among the oldest young children. In that group, a similar proportion of children, 31 and 35%, respectively, consume dark green and deep yellow vegetables.

Table 5 shows the average amount of the top five vegetables consumed. White potatoes were less commonly consumed. However, other vegetables, including Chinese cabbage and tomatoes, became the number one type of vegetable consumed by 6 months and were consumed increasingly with age, with up to 76% of young children aged 30–35 months reporting other vegetables. Chinese cabbage was the single most commonly consumed vegetable among all infants and young children with the exception of the youngest infants (Table 5). Carrots and spinach were the second or third most common vegetables among infants aged 6–11 months; for those aged between 12 and 23 months, carrots were replaced by tomatoes. Among young children aged 24–35 months, the top three vegetables were Chinese cabbage, carrots, and tomatoes (Table 5).

### Meat and other protein sources

At age 6–8 months, 74% of infants consumed some type of non-milk protein sources and the proportion increased to 92% between 9 and 11 months. By 12 months of age, nearly all children consumed some type of meat or other protein sources a day (Table 7). The average amount of the top five protein sources consumed is shown in Table 2.

**Table 7:** Percentage of infants and young children consuming meat or other protein sources at least once a day in the Maternal Infant Nutrition Growth (MING) study

	Age group							
	
Food/food groups	6–8 months (*n =* 201)	9–11 months (*n =* 235)	12–14 months (*n =* 125)	15–17 months (*n =* 75)	18–20 months (*n =* 160)	21–23 months (*n =* 110)	24–29 months (*n =* 248)	30–35 months (*n =* 196)
Any meat or protein source	74	92	97	97	99	98	98	99
Non-baby food meat	20	45	54	51	71	67	74	84
Types of meat								
Pork/ham	12	25	34	25	48	55	54	62
Chicken or duck	1	3	7	5	9	8	12	15
Fish, shrimp or shellfish	7	17	15	20	27	12	23	27
Beef	1	2	5	4	4	3	8	19
Hot dogs/sausages	0	1	3	7	3	3	6	3
Lamb	1	0	0	1	3	1	1	2
Organ meat	3	4	5	5	3	2	2	2
Other^a^	0	0	1	0	0	1	0	1
Baby food meat	1	1	1	0	0	0	0	1
Other protein sources	71	89	94	95	98	96	97	97
Dried beans and meat								
substitutes^b^	3	7	12	20	16	12	20	20
Eggs	51	62	65	60	66	69	69	77
Peanut butter, nuts, seeds	0	2	9	0	7	9	8	8
Cheese	1	0	1	0	0	1	1	2
Yogurt	1	1	2	5	7	15	9	17
Beans^c^	1	1	4	4	6	4	5	3
Soup^d^	5	6	17	11	10	14	15	9

a Includes rabbit meat, goose, and pigeon meat.

b Includes black soybean, soybean, and soybean products including tofu.

c Includes mung beans, red beans, kidney bean, broad bean, lentils, pea, and green soy bean.

d The amount of protein provided by soup varies.

Among animal protein sources, eggs were the leading source with a percentage of 51% at age 6–8 months reporting eggs (Table 7). The proportion increased steadily with age to 77% at age 30–35 months. Pork or ham was the second most commonly consumed animal protein: 12% for 6- to 8-month-old infants and 25% for 9–11-month-old infants, increasing to 62% by age 30–35 months. Fish, shrimp and shellfish were also commonly consumed animal protein sources. The proportion of children consuming poultry was low, only 7–15% of young children (Table 7). Beef was another type of meat that was not commonly consumed, with the exception of 19% among the young children aged 30–35 months. Yogurt and cheese were rarely consumed among infants and consumed slightly more often among young children (Table 7).

Among non-animal protein sources, dried beans and meat substitutes (including tofu) were not commonly consumed among infants but they were consumed more in young children with a percentage up to 20% among the young children aged 30–35 months. Peanut butter, nuts and seeds were consumed by 8–9% of young children.

### Desserts/sweets, sweetened beverages, and salty snacks

From 6 to 8 months onwards, the children began to consume some type of desserts/sweets with a percentage of 14% at 6–8 months to about a quarter by 11 months (Table 8). The proportion increased to more than one-third of children by 12–14 months, and at age 21–35 months, over 40% of the children consumed at least one type of sweets in a day.

**Table 8:** Percentage of infants and young children consuming desserts, sweets, sweetened beverages, and salty snacks at least once a day in Maternal Infant Nutrition Growth (MING) study

	Age group							
	
Food/food groups	6–8 months (*n =* 201)	9–11 months (*n =* 235)	12–14 months (*n =* 125)	15–17 months (*n =* 75)	18–20 months (*n =* 160)	21–23 months (*n =* 110)	24–29 months (*n =* 248)	30–35 months (*n =* 196)
Any type of dessert, sweet, sweetened beverage	14	23	38	37	33	44	44	42
Desserts and candy	11	22	29	32	26	36	36	26
All cakes, pies, cookies, and pastries	11	22	29	31	25	36	33	20
Baby cookies, teething biscuits	1	0	0	0	0	0	0	0
Other cookies	10	20	21	24	19	31	24	13
Cake	2	2	8	3	3	2	6	5
Pies and pastries	0	0	0	1	1	0	0	1
Ice cream, pudding	0	0	0	0	1	3	1	2
Other desserts^a^	1	0	1	3	3	5	4	2
Candy	0	0	2	1	3	3	4	8
Other sweets	3	2	11	8	8	11	14	18
Milk flavoured sweets	0	1	6	4	4	7	7	9
Sugar, syrup, honey, preserves	3	1	5	4	4	4	9	10
Sweetened beverages	1	1	4	1	3	3	0	5
Carbonated sodas	0	0	0	1	0	1	0	1
Fruit-flavored drinks	1	1	3	0	3	2	0	3
Other^b^	0	0	1	0	1	0	0	1
Salty snacks^c^	1	2	1	1	3	6	3	3

a Includes tea soup, glue pudding, tortoise jelly, caramel treats, mung bean cake (green bean cake), and laozao (fermented sticky rice desert).

b Includes tea (all types).

c Includes potato chips, popcorn, and other types of chips and salty snacks.

The most commonly consumed desserts were cookies (with an average amount ranging from less than 1 g among infants to 2–4 g among young children). Other sweets, including milk-flavoured sweets, sugar, syrup, honey, and preserves, were the next commonly consumed desserts/sweets. Sweetened beverages (e.g. carbonated sodas, fruit-flavoured drinks or tea with added sugar) and salty snacks were very rarely consumed.

## Discussion

To our knowledge, this was the first study to characterise in detail the food consumption patterns of infants and young children in China. The results identified some positive aspects of the diet and also areas for improvement, as discussed below.

### Milk consumption

Breastfeeding rates were lower than recommended. Only about 58% of infants 6–8 months and 35% of infants 9–11 months received any breast milk. This is lower than infants from rural counties in the central and western provinces in China, where 55.5% were breastfed for up to 1 year (32). Breast milk is the ideal source of infant nutrition because of its nutritional, immunological, and psychological benefits (33), as well as the long-term benefits to metabolism and protection against disease later in life (34). Infants should be exclusively breastfed for the first 6 months of life and thereafter receive nutritionally adequate and safe complementary food while breastfeeding continues for up to 2 years or beyond as recommended by the World Health Organization (WHO) (35) and the Chinese Nutrition Society (36).

Despite WHO and Chinese Nutrition Society recommendations on breastfeeding, the use of infant formula among infants and growing-up milk among young children are common practices in China. Infant formula is a product based on cows’ milk that is formulated to make it suitable as the sole source of nutrition for infants (37). Growing-up milk is a milk product fortified with minerals and vitamins intended for young children aged 1–3 years. Growing-up milk can help to increase the dietary intake of key nutrients such as iron, zinc, vitamin D, and vitamin C in young children (38, 39). Indeed, we also found in our previous analysis that infant formula and growing-up milk provided the number one source of iron and zinc among the infants aged 6–12 months and young children aged 12–24 months in the MING study, contributing 27%–31% of total iron intake, for example (29).

### Fruit and vegetable consumption

We found that the consumption of fruits and vegetables among the infants and young children in this study was not optimal, especially among infants. About half of the infants aged 6–11 months did not consume any fruits and vegetables in the day of dietary recall. The consumption of vegetables among young children was higher than among infants, but overall there were still a substantial proportion of young children who did not consume any vegetables or fruits on the day of the recall. It is recommended that once infants begin consuming solid foods, the goal is gradually to include a variety of vegetables and fruits on a daily basis as part of a healthy diet (40, 41). The choice of vegetable is also important because dark green leafy vegetables and deep yellow vegetables are more nutrient dense than less highly coloured vegetables. Only about one-third of the children in this study reported consuming dark green leafy vegetables, and the consumption of deep yellow vegetables was even lower. The most commonly consumed vegetable was Chinese cabbage, which is a pale colour vegetable with light green leaves. Therefore, the nutritive value of this vegetable is limited. In this population, more emphasis should be placed on the consumption of dark green leafy and deep yellow vegetables, and colourful fruits because they are good sources of many vitamins (such as vitamins A, C, and K, and folate) and minerals.

Studies in the United States and Brazil have also reported less-than-adequate fruit and vegetable intake among infants and young children (18, 21, 42). Early exposure to fruits and vegetables has been related to children's preference for and consumption of these foods throughout childhood (43, 44). Although many factors can influence food choices, a foundation for healthy food habits can be created in childhood (45). A diet high in fruits and vegetables has been shown to be associated with reduced risk of several chronic diseases later in life (46). It is therefore important to emphasise to parents the fundamental role that fruits and vegetables play in a healthy diet and encourage them to expose their children to a variety of fruits and vegetables early (47).

### Infant cereal and other grains

We found that infant cereal was not commonly consumed in this population; instead, from the age of 6 months, rice became the predominant grain-based food across all age groups. Results from our previous study showed that rice was the third source of energy (after infant formula and breast milk) among infants aged 6–11 months, second among younger children, and first among older children (29). In addition to rice, noodles were another grain product commonly consumed from 9 months of age and onward. The low consumption of infant cereal among the infants is of concern given the risk of inadequate intake of iron in this age group (27).

Dietary iron prevents iron deficiency and iron deficiency anaemia and supports cognitive and motor development during infancy (48, 49). Infant cereal is a food that meets this need and helps to provide iron during the transition from an all-milk diet to one that includes meat. In addition, infant cereal is also an important source of zinc and other nutrients. In China, the consumption of iron-fortified infant cereals (made from different grains, including rice, wheat, and oats) is one of the several strategies for preventing iron deficiency anaemia recommended by the Chinese Nutrition Society and China Medical Association in China (36, 40).

### Meat and protein sources

Eggs were the most commonly consumed non-milk protein source in this population, and pork was the most frequently reported meat. Red meats such as beef and lamb are good sources of high-quality protein and are also rich in iron, zinc, and other micronutrients. The iron in red meats is heme iron, which is more easily absorbed by the body (50). Beef and lamb, however, were infrequently consumed. In addition, less than one-fifth of children consumed fish, shrimp, or shellfish.

### Desserts/sweets

Compared with counterparts in the United States and European countries, the proportion of young children consuming any desserts/sweets was low (18, 21, 42, 51, 52). For example, the percentage of young children consuming any desserts/sweets was 62–80% in Feeding Infants and Toddler study in the United States, whereas it was only 33–43% in this study. Another finding is that the consumption of sweetened beverages in this population was very rare. However, in light of the poor-nutrient density and high-energy density of many desserts and sweets and the rapidly increasing prevalence of childhood obesity in China (53), the consumption of desserts/sweets should be closely monitored. Indeed, previous studies in China have already demonstrated that during 1991–2004, the consumption of desserts, sweetened beverages, and other snacks increased markedly among children and adolescents aged 3–17 years in China (54, 55). In 2015, the WHO published a guideline on sugar intake, responding to the concern about the high-level intake of free sugars in many Western countries and the associated poor dietary quality, obesity, and risk of non-communicable diseases (56).

### Limitation of the study

A number of limitations should be considered when interpreting the findings of the study. First, all children were recruited from the MCCC in selected cities in China, including the most industrialised cities, Beijing, Shanghai, and Guangzhou. The incomes of the families who participated in the study tended to be higher than the national averages. Therefore, it is an urban rather than a nationally representative sample. Indeed, it has been reported that the proportion of young children consuming meat or milk is higher in urban than in rural areas (57). Second, the information on food consumption patterns was based on one 24-h dietary recall. Though this is appropriate for evaluating diet patterns (58), random errors due to day-to-day variations in individual diets could lead to over- or underestimations. In addition, it is possible that for some children the recall day was not typical of their usual dietary pattern.

## Conclusions

This study characterised in detail the food consumption patterns of infants and young children from urban areas of China. Some positive aspects of the diet were found, including a high proportion of children consuming nutrient-fortified milk, infant formula, and growing-up milk, and a low proportion of children consuming sweetened beverages. Areas for improvement were identified: 1) continued breastfeeding among infants and young children was low; 2) the consumption of fruits and vegetables, particularly dark green vegetables, was low for all ages; 3) iron-rich foods, including infant cereal and red meats, were not commonly consumed among infants, while iron-poor rice or noodles were heavily consumed. The findings of this study provide important insight for developing detailed food consumption guidelines for this age group, especially for encouraging continued breastfeeding after first 6 months, the daily consumption of fruits and vegetables, the use of fortified infant cereals during weaning, and the increased consumption of food sources of iron, such as lean red meats.
